# Simultaneous Separation and Purification of Five Polymethoxylated Flavones from “Dahongpao” Tangerine (*Citrus tangerina* Tanaka) Using Macroporous Adsorptive Resins Combined with Prep-HPLC

**DOI:** 10.3390/molecules23102660

**Published:** 2018-10-16

**Authors:** Zhenqing Li, Ziyan Zhao, Zhiqin Zhou

**Affiliations:** 1 College of Horticulture and Landscape Architecture, Southwest University, Chongqing 400716, China; zhenqinglee@126.com(Z.L.); jlljtsy@163.com(Z.Z.); 2 The Southwest Institute of Fruits Nutrition, Liangjiang New District, Chongqing 401121, China; 3 Key Laboratory of Horticulture Science for Southern Mountainous Regions, Ministry of Education, Chongqing 400715, China

**Keywords:** *Citrus* flavonoids, PMFs, UPLC-Q-TOF-MS/MS, prep-HPLC, HPD 300 resin, new method

## Abstract

In this study, a preparative separation method was established to simultaneously isolate the polymethoxylated flavones (PMFs) from the peel of “Dahongpao” tangerine using macroporous adsorptive resins (MARs) combined with prep-HPLC. The total PMFs were enriched using MARs to remove most sugars, water-soluble pigments, and flavanones, and the eluents obtained were analyzed by ultra-performance liquid chromatography (UPLC) to determine the PMF composition. The separation and purification of PMFs were carried out by using a mass spectrometry-guided prep-HPLC with a gradient elution of acetonitrile-water (*v*/*v*), simultaneously. The purity of these PMFs was determined by UPLC, and their chemical structures were confirmed by electrospray ionization mass spectrometry (ESI-MS-MS), ultraviolet (UV), and nuclear magnetic resonance (NMR). Using the present method, five PMFs, including 5,6,7,4’-tetramethoxyflavone (1), nobiletin (2), tangeretin (3), sinensetin (4), and 5-hydroxy-6,7,8,3’,4’-pentamethoxyflavone (5), can be purified simultaneously, and the purity of the compounds obtained were 95.3%, 99.7%, 99.5%, 98.9%, and 98.1%, respectively. The method reported here is simple, rapid, and efficient, and it can be used to separate PMFs from citrus fruit peels and, potentially, other plant materials.

## 1. Introduction

Mandarin (*Citrus reticulata* Blanco) is one of the most important cultivated species in the genus *Citrus* L., among them “Dahongpao” tangerine (*Citrus tangerina* Tanaka) was widely cultivated in southwestern China early 1980s. In the past decades, as a fresh eaten fruit, “Dahongpao” tangerine has been almost completely replaced by various hybrid mandarins in China, but the health promotion or medicinal values of “Dahongpao” tangerine have attracted more and more attention from researchers [1,2,3]. Since the dried peel of “Dahongpao” is a major source of Guang Chenpi, also called *Pericarpium Citri Reticulatae* in pharmacology, which has been a traditional Chinese medicine since ancient times for curing allergy, as an antitussive, expectorant, and so forth [4,5,6,7]. As one of the precious citrus germplasms, the plants of “Dahongpao” tangerine are now mainly maintained in Citrus Research Institute of CAAS (Chongqing, China) and Wanzhou, Chongqing, China.

Polymethoxylated flavones (PMFs) are a subgroup of citrus flavonoids which usually have four or more methoxy groups (OCH_3_) on their basic skeleton [8]. Until now, there are at least 135 PMFs compounds reported in the current literature. Study of PMFs has increasingly become the focus of researchers in recent years [9,10]. Current reports suggest that the PMFs possess a wide variety of bioactivities, including anti-inflammatory, antioxidant [10], antimicrobial [11], anticancer [12], glucolipid metabolism-regulating, and others [13]. More importantly, PMFs were gradually used to prevent and treat various human chronic diseases [14,15,16]. Therefore, it was of great importance to establish an effective, simple, rapid, and economical method to separate and purify different PMF compounds from citrus materials, especially those rich in PMFs, for better use of citrus germplasm. Although the separation and purification of citrus PMFs have been widely studied in previous literature, the most recently reported method still faced problems such as that it is difficult to separate compounds with highly similar structures [17]. Therefore, a deep study on the isolation and purification of PMFs is still needed. 

Silica gel column chromatography was the traditional separation and purification method of flavonoids from citrus fruits [18]. It was a mature but complicated method with tedious steps. In addition, the mobile phase in gradual elution mode used many different organic solvents, which was both costly and environmental unfriendly, because the mobile phase could not be recycled. Recently, high speed counter-current chromatography (HSCCC) was used to separate and purify flavonoids from fruits [19], but it was difficult to choose the two-phase solvent system, due to lack of system directions. Compared with the methods mentioned above, prep-HPLC is effective, rapid, versatile, and usually stable [20]. However, there were also problems with prep-HPLC methods, for example, it is difficult to achieve great isolation of PMFs from crude extract because of the complexity in the establishment of liquid phase separation gradients. In addition, more impurities that were present in the crude extract were a significant threat to the stationary phase, which is liable to be contaminated, and the column is easily clogged. Therefore, many techniques, including solute/solvent distribution, silica gel column chromatography, macroporous adsorption resins (MARs), and polyamide resins, have been used to remove impurities and purify the target components.

Among the current pre-purification techniques, MAR is a porous polymer material used to selectively adsorb components from aqueous and non-aqueous systems by electrostatic forces, hydrogen bonding interactions, complexation and size screening actions, and more. [21]. Recently, MAR has captured much attention because of its high capacity of absorption and desorption, easy regeneration, simple techniques, and low cost. Most of the impurities can be removed from crude extractions and some target compounds can be enriched by MARs, which makes it be widely used in medicine industry [22].

Therefore, the aim of this work was to establish a highly efficient method for simultaneous isolation and purification of PMFs from the peel of “Dahongpao” tangerine. MARs were used to remove impurities, especially flavanones, in crude extract, UPLC was used to determine the composition of PMFs, a MS-directed prep-HPLC was to isolate and purify the PMFs, and MS, UV and NMR analysis were used to confirm their chemical structures. Five PMFs, 5,6,7,4′-tetramethoxyflavone, nobiletin, tangeretin, sinensetin, and 5-hydroxy-6,7,8,3′,4′-pentamethoxyflavone, were obtained in one step using our method, and the purities of the compounds were all above 95%. Our study is of great importance for future use of citrus germplasms, both in China and other countries.

## 2. Results and Discussion

### 2.1. Static Adsorption and Desorption Capacities

To have a simple sample cleaning-up method for prep-HPLC system, eight resins (in Table 1) were tested for adsorption and desorption capacities. The results were shown in Table 2.

The adsorption capacities of HPD 300, AB-8, and HPD 100 resins toward total PMFs of tangerine peels were significantly higher than those of other resins. However, the desorption capacity and desorption rate of AB-8 resin were the lowest in these three resins. On the contrary, although the desorption rate of HPD 300 resin was not the highest, the adsorption and desorption capacity were the highest, which may correlate with the surface areas (800–870 m^2^/g), the polarity (weak polar), and the average pore dimeter (50–55 Å) of the HPD 300 resin. Therefore, HPD 300 resin was selected to further investigate the adsorption process. It is generally known that PMFs consist of non-polar phenyl groups and polar multi-hydroxyl groups [3,23] and, according to the “like dissolve like” rule [24,25], the nonpolar, weak polar, and polar resins were applicable to the adsorption of total PMFs. In addition, the HPD 300 resin with larger specific surface area and smaller average pore dimeter possessed a slightly stronger adsorption and desorption capacity than the other resins, suggesting that the specific surface area and average pore dimeter might be two of the critical factors that affect the desorption process [26,27,28].

### 2.2. Adsorption Kinetics

**Figure 1:**
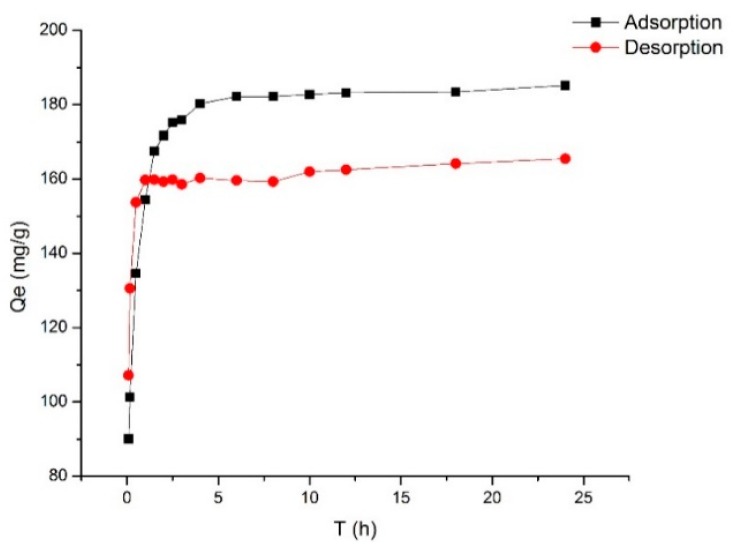
Adsorption kinetics curve and desorption kinetics curve for total PMFs on HPD 300 resin.

The kinetics of adsorption that describes the solute uptake rate governing the contact time of the sorption reaction, is one of the important characteristics that define the efficiency of sorption [23,29]. Hence, in the present study, the adsorption kinetics of the total PMFs was determined to understand the adsorption behavior of HPD 300 resin. The static kinetic curve obtained was shown in Figure 1.

As shown in Figure 1, the adsorption process exhibited three stages. In the first stage, the adsorption capacity showed a linear and rapid increase over the first 2.5 h, but in the second stage, the adsorption capacity of HPD 300 resin increased slowly and reached adsorption equilibrium at 6 h. In addition, in the process of static desorption, it was desorbed rapidly and reached equilibrium within first 1 h. Thus, HPD 300 resin was the most suitable resin for the enrichment of total PMFs.

To elucidate the adsorption behavior and mechanism of HPD 300 resin, pseudo-first order, pseudo-second order, and particle diffusion kinetics models, were adopted to evaluate the adsorption process. The equations and parameters of the three kinetic equations are summarized in Table 3.

The adsorption process of total PMFs on HPD 300 resin was elaborated by the pseudo-second order kinetics model, and a result with a good correlation (*R*^2^ = 0.9999) was obtained. The theoretical maximum adsorption capacity Q_e_ calculated by the pseudo-second order model, was very close to the experimental data. The results showed that the adsorption rate was limited by chemisorption, and the kinetics for the entire adsorption process of total PMFs on HPD 300 resin was reasonably well explained by the pseudo-second order equation. Although the intra-particle diffusion kinetics models could not describe well the whole adsorption process of the total PMFs in this study (*R*^2^ ranges from 0.6008 to 0.9660), the process could be divided into two stages. 

Generally, the adsorption capacity increased transiently with adsorption time, before reaching the adsorption equilibrium [30,31]. The results obtained in this study were in agreement with the previous literature. The respective linear correlation of intra-particle diffusion kinetics models did not pass through the origin, which indicated that intra-particle diffusion was not the only rate-limiting step in the adsorption processes [32,33]. The adsorption could be driven by multi-diffusion steps, including film diffusion (transport of adsorbates from boundary film to resin surface) and intra-particle diffusion (transport of adsorbates within resin pores) [34,35]. The adsorption of HPD 300 resin on total PMFs may be driven by film diffusion.

### 2.3. Adsorption Isotherms

**Figure 2:**
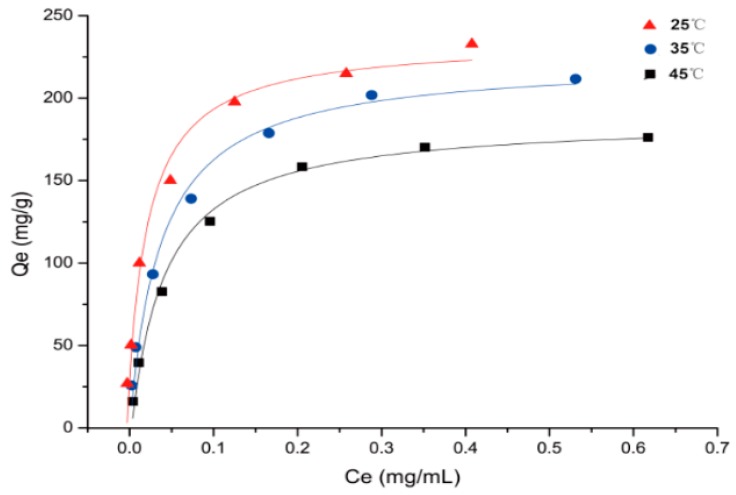
Adsorption isotherms of total PMFs on HPD 300 resin at 25, 35, and 45 °C.

To investigate the isothermal adsorption properties of HPD 300 resin. The adsorption isotherms, equations, and parameters of PMFs on HPD 300 resin at 25, 35, and 45 °C were suggested in Figure 2 and Table 4. 

The adsorption capacity of PMFs on HPD 300 resin increased rapidly at lower concentrations of extract, and then reached the saturation plateau. However, as the temperature was increased from 25 to 45 °C, the adsorption capacity of PMFs decreased, especially at 45 °C, which was significantly lower than that at 25 °C. The results revealed that the adsorption process of PMFs on HPD 300 resin was intrinsically exothermic [36].

The higher correlation coefficients (*R*^2^) indicated that Langmuir model was more suitable for describing the tested adsorption process within the concentration range. Langmuir isotherm model described the monolayer adsorption on a homogeneous surface with no interaction between adjacent adsorbed molecules [37]. The value of the Langmuir constant 1/K_L_ indicates whether the isotherm is favorable (0 < 1/K_L_ < 1), or linear (1/K_L_ = 1), or unfavorable (1/K_L_ > 1) [38]. The values of 1/K_L_ in Table 4 were all between 0 and 1, indicating that the isotherm of HPD 300 resin was favorable.

### 2.4. Dynamic Adsorption and Desorption

**Figure 3:**
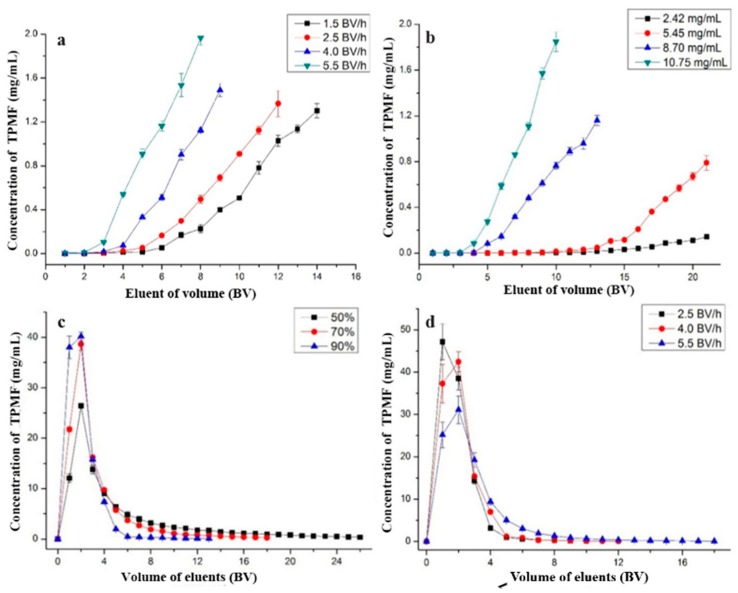
Dynamic breakthrough curve (**a**,**b**) and dynamic desorption curves (**c**,**d**) of PMFs on column.

Dynamic adsorption and desorption studies were carried out with different feeding speeds, initial concentrations of PMFs, ethanol concentrations, and eluting speeds. The results were shown in Figure 3.

#### 2.4.1. Optimal Feeding Speed

As shown in Figure 3a, with an increase in feeding speed, the concentration of total PMFs in eluent solution was increased. The loading amounts under these speeds were 139.75, 118.25, 86.00, and 64.50 mg, respectively. It can be seen that the loading time exceeded 4 h when the feeding speeds were 1.5 BV/h and 2.5 BV/h; when the feeding speed was 5.5 BV/h, the leakage was rapidly appeared, but the loading amount was not satisfactory. Considering the production efficiency, 4.0 BV/h was selected as the optimal feeding speed.

#### 2.4.2. Optimal Concentration of Total PMFs

When the feeding speed was 4.0 BV/h, the concentration of total PMFs was 2.42 mg/mL, and the leakage did not appear until loading volume was 21 BV. While, at other concentrations (5.45, 8.70, and 10.75 mg/mL), the leakage appeared at 19 BV, 11 BV, and 8 BV, and the loading amounts on the resin were 103.55, 95.70, and 85.60 mg, respectively, in Figure 3b. With an increase in the concentration, the leakage appeared earlier, but loading amount decreased. Comprehensively, 8.70 mg/mL was selected as the optimal loading concentration.

#### 2.4.3. Impurities Removal

In order to remove sugar, water-soluble pigments and a small amount of flavanone adsorbed by the resin at the same time, 7% ethanol solution was used, and the elution speed was 4 BV/h. The color of the solution (1–4 BV) after eluting was light yellow, and lightened at 5 BV, approaching colorless at 6 BV. The flavanone was eluted with the eluent at 2–5 BV detected by UPLC, but few PMFs were detected. 

#### 2.4.4. Optimal Desorption Ethanol Concentration 

Ethanol solution was a suitable desorbent, due to its easy removal and nontoxicity. As shown in Figure 3c, total PMF solutions were desorbed with ethanol solutions, the desorption capacity increased gradually with an increase of ethanol concentration. When the concentration of ethanol (50%, 70%, and 90%) was increased, the total PMF in solution was rapidly desorbed, while the desorption volumes were 23, 8, and 4 BV, respectively. Considering the economic efficiency and eco-friendliness, 90% ethanol was selected as the desorption solution in the dynamic desorption experiments.

#### 2.4.5. Optimal Eluting Speed

It can be found in Figure 3d that the desorption process of HPD 300 resin at different eluting speeds (2.5, 4.0, and 5.5 BV/h) reached their plateaus while the elution volumes were 3, 4, and 14 BV, respectively. It was clear that when the eluting speed on HPD 300 resin increased, the desorption time increased, as a result of efficiency and economy, and 4.0 BV/h was selected as the optimal desorption eluting speed.

### 2.5. Effect of Enrichment by HPD 300 Resin on PMFs Extraction 

**Figure 4:**
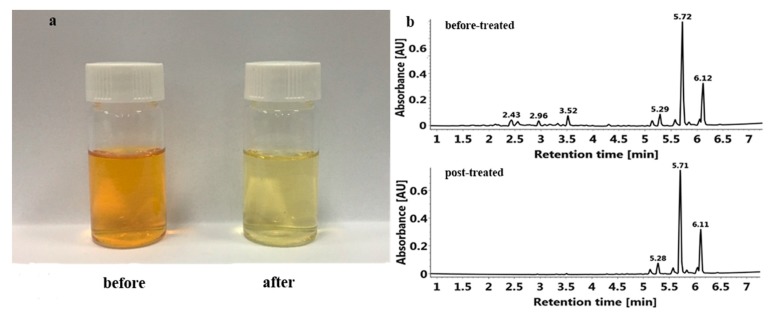
Effects of before- and post-enrichment on PMF extraction. (**a**) PMF extract solution before and after purification by HPD 300 resin; (**b**) Effects of HPD 300 resin on UPLC spectrum of PMFs extract.

To investigate the enrichment and purification effect of MARs, the composition and content of PMFs in the crude extracts before and after enrichment on HPD 300 resin were determined; detailed information is listed in Table 5 and Figure 4.

The results in Table 5 showed that the components of PMF in the crude extracts did not change after being treated by HPD 300 resin, but the total PMF content increased from 31.720 ± 1.255 mg/g to 594.57 ± 28.05 mg/g, which was higher than before enrichment by about 18.74-fold. The content of each PMF component was greatly increased, among which, 5,7,4′-trimethoxyflavone increased the most, its content after enrichment was increased 87.89-fold than that before enrichment.

Although the crude extract solution and the purified total PMF solution were under the same concentration (in Figure 4a), the former has a dark color but the latter was light and clear because most impurities (e.g. water-soluble pigments) were removed. In Figure 4b, the crude extract of PMFs before purification contained other flavonoids (1.5–4.5 min), such as flavanones, which were significantly reduced after purification. Thus, HPD 300 resin could effectively enrich PMFs and remove impurities.

### 2.6. Separation and Purification of PMFs

#### 2.6.1. Optimal Conditions of Prep-HPLC

**Figure 5:**
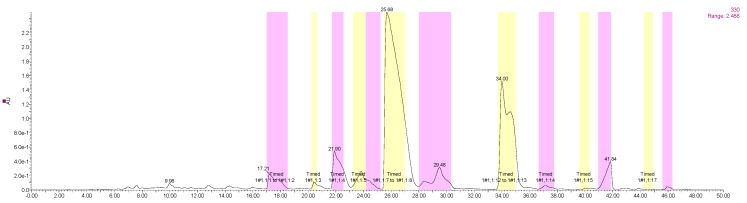
Collection conditions under automatic fraction trigger.

To isolate and purify the PMFs compounds simultaneously, a MS-directed prep-HPLC method needed to be established. Based on the literature and studies, a XBridge C18 column (19 × 250 mm, 5 μm) was selected, and other conditions, such as the acid/buffer salt, flow rate of mobile phase, injection volume, elution gradient, and collection time, were optimized as follows: acetonitrile–water (A) with 0.1% formic acid (B) was used as the mobile phase with a gradient elution (0–6 min, 18–30% A; 6–35 min, 30–42% A; 35–45 min, 42–60% A; 45–50 min, 60%–18% A), the flow rate was 20 mL/min, injection volume was 650 μL. Peak fractions were collected according to the retention time and the online-QDa detection. The collection conditions were showed in Figure 5.

Each prep-HPLC peak fraction was automatically collected, and the same fractions were combined and concentrated in vacuo. Finally, five PMF monomers were obtained after lyophilization.

#### 2.6.2. Identification of Isolated PMF Monomers

**Figure 6:**
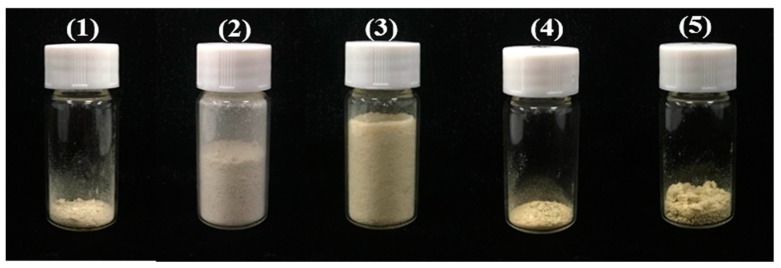
Five PMFs monomers obtained in this study. (**1**) 5, 6, 7, 4’-tetramethoxyflavone (94.8 mg); (**2**) nobiletin (1228.3 mg); (**3**) tangeretin (528.3 mg); (**4**) sinensetin (131.8 mg); (**5**) 5-hydroxy-6,7,8,3′,4′-pentamethoxyflavone (125.0 mg).

**Figure 7:**
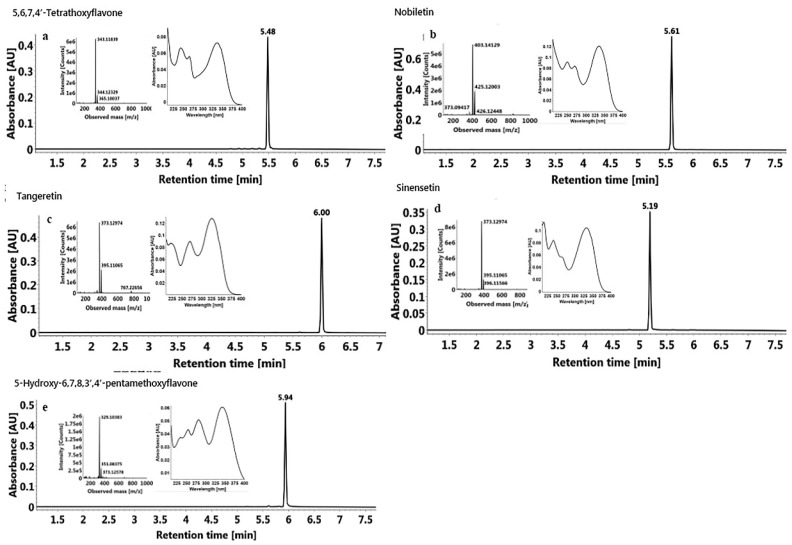
UPLC chromatograms, UV, and MS spectrums of five PMFs isolated by prep-HPLC.

The obtained compounds (Figure 6 and Figure 7) were identified by UV, MS, and NMR, and compared with standards and the literature. The MS and NMR data for these compounds were as follows: 

Compound 1 (Figure 7a, 94.8 mg) was a yellow powder: ESI-MS *m*/*z* 343. 33839 [M + H]^+^, C_19_H_18_O_6_. ^1^H-NMR (400 MHz, CDCl_3_) *δ* 7.93–7.85 (m, 2H), 7.06–6.97 (m, 2H), 6.60 (s, 1H), 6.44 (s, 1H), 4.01 (s, 3H), 3.99 (s, 3H), 3.96 (s, 3H), 3.92 (s, 0H), 3.91 (s, 0H), 3.89 (s, 3H), 3.83 (d, *J* = 1.9 Hz, 0H). ^13^C-NMR (101 MHz, CDCl_3_) *δ* 177.94(C-4), 162.18(C-2), 160.76(C-4’), 156.48(C-7), 156.29(C-5), 151.95(C-9), 130.79(C-6), 127.70(C-2’), 123.83(C-1’), 114.45(C-5’), 108.99(C-10), 106.90(C-3), 92.60(C-8), 61.56, 56.55, 56.29, 55.47 (4 × OMe) (Figure S1). The above data were in accordance with those reported in the literature, so it was identified as 5,6,7,4’-tetramethoxyflavone [39], and the purity detected by UPLC was 95.3%. 

Compound 2 (Figure 7b, 1228.3 mg) was a white powder: ESI-MS *m*/*z* 403. 14129 [M + H]^+^, C_21_H_22_O_8_. ^1^H-NMR (400 MHz, CDCl_3_) *δ* 7.58 (dd, *J* = 8.4, 2.0 Hz, 1H), 7.42 (d, *J* = 2.0 Hz, 1H), 7.00 (d, *J* = 8.5 Hz, 1H), 6.66 (s, 1H), 4.11 (s, 3H), 4.03 (s, 3H), 3.98 (s, 3H), 3.97 (s, 3H), 3.96 (s, 6H). ^13^C-NMR (101 MHz, CDCl_3_) *δ* 177.34(C-4), 161.13(C-2), 151.99(C-4’), 151.46(C-7), 149.32(C-3’), 148.41(C-5), 147.71(C-9), 144.12(C-6), 138.00(C-8), 123.99(C-1’), 119.67(C-6’), 114.78(C-10), 111.27(C-5’), 108.63(C-2’), 106.81(C-3), 62.26, 61.96, 61.81, 61.66, 56.09, 55.98(6 × OMe) (Figure S2). These data were in agreement with the values in previous literature, so it was identified as nobiletin [40]; the purity detected by UPLC was 99.7%. 

Compound 3 (Figure 7c, 528.3 mg) was a light yellow powder: ESI-MS *m*/*z* 373.12974 [M + H]^+^, C_20_H_20_O_7_. ^1^H-NMR (400 MHz, CDCl_3_) *δ* 7.93–7.84 (m, 2H), 7.07–6.98 (m, 2H), 6.62 (s, 1H), 4.11 (s, 3H), 4.03 (s, 3H), 3.95 (s, 6H), 3.89 (s, 3H). ^13^C-NMR (101 MHz, CDCl_3_) *δ* 177.29 (C-4), 162.30 (C-2), 161.21 (C-4’), 151.38 (C-7), 148.38 (C-5), 147.72 (C-9), 144.08 (C-6), 138.08 (C-8), 127.71(C-2’, 6’), 123.83(C-1’), 114.85(C-10), 114.51(C-3’, 5’), 106.65(C-3), 62.24, 62.01, 61.81, 61.64, 55.49 (5 × OMe) (Figure S3). These data agreed well with those reported in the literature, so it was identified as tangeretin [41]; the purity detected by UPLC was 99.5%.

Compound 4 (Figure 7d, 131.8 mg) was a yellow powder: ESI-MS *m*/*z* 373.32974 [M + H]^+^, C^20^H^20^O^7^. ^1^H-NMR (400 MHz, CDCl_3_) *δ* 7.55–7.48 (m, 1H), 7.33 (s, 1H), 7.28 (s, 0H), 6.97 (d, *J* = 8.5 Hz, 1H), 6.81 (s, 1H), 6.62 (s, 1H), 4.00 (s, 6H), 3.98 (s, 3H), 3.96 (s, 3H), 3.93 (s, 3H). ^13^C-NMR (101 MHz, CDCl_3_) *δ* 177.25(C-4), 161.18(C-2), 157.69(C-7), 154.49(C-5), 152.57(C-9), 151.84(C-4’), 149.28(C-3’), 140.37(C-6), 124.09(C-1’), 119.60(C-6’), 112.83(C-10), 111.16(C-5’), 108.71(C-2’), 107.33(C-3), 96.25(C-8), 62.17, 61.52, 56.31, 56.13, 56.07(5 × OMe) (Figure S4). The above data were consistent with the literature reported previously [42], so it was identified as sinensetin; the purity detected by UPLC was 98.9%. 

Compound 5 (Figure 7e, 125.0 mg) was a yellow powder: ESI-MS *m*/*z* 389.38383 [M + H]^+^, C_20_H_20_O_8_. ^1^H-NMR (400 MHz, CDCl_3_) *δ* 12.54 (s, 1H), 7.59 (dd, *J* = 8.6, 2.1 Hz, 1H), 7.42 (d, *J* = 2.2 Hz, 1H), 7.00 (d, *J* = 8.5 Hz, 1H), 6.62 (s, 1H), 4.12 (s, 3H), 3.99 (s, 6H), 3.98 (s, 3H), 3.96 (s, 3H), 1.32 (s, 1H). ^13^C-NMR (101 MHz, CDCl_3_) *δ* 182.95(C-4), 163.91(C-2), 152.98(C-7), 152.48(C-4’), 149.53(C-3’), 149.39(C-9), 145.75(C-5), 136.58(C-6), 132.95(C-8), 123.70(C-1’), 120.14(C-6’), 111.29(C-5’), 108.80(C-2’), 106.99(C-10), 103.98(C-3), 62.05, 61.71, 61.11, 56.12, 56.00 (5 × OMe) (Figure S5). These data were consistent with the literature reported previously [42], so it was identified as 5-hydroxy-6,7,8,3’,4’-pentamethoxyflavone, and the purity detected by UPLC was 98.1%.

## 3. Materials and Methods 

### 3.1. Plant Materials and Reagents

“Dahongpao” tangerine (*Citrus tangerina* Tanaka) was selected as the plant material harvested in Wanzhou, Chongqing, China at the ripening stage, and then transported to laboratory as soon as possible. After washing by tap water, the fruits were manually peeled, and the peel was put into the air drier at 50 °C until achieving constant weight. Then, they were ground into powder (60 mesh).

The main reagents used in this study were as follows: methanol and ethanol (analytical grade) were purchased from Kelong Chemical Reagent Factory, Chengdu, China; hydrochloric acid (analytical grade) and sodium hydroxide (analytical grade) were purchased from Chuandong Chemical Co., Ltd. (Chongqing, China); formic acid (MS grade), DMSO (LC grade), and CDCl_3_ (NMR grade) were purchased from Sigma-Aldrich, St. Louis, MO, USA; methanol (MS grade) used for UPLC was purchased from Fisher, and distilled water was made by Milli-Q Advantage A10 (Millipore Co., Ltd., USA). The detailed information of the main PMF standards used in this study are listed in Table 6.

MARs, including D 101, HPD 100, HPD 300, DM 130, HPD 400, and HPD 600, were purchased from Baoen Adsorption Material Technology Co., Ltd. In Cangzhou (Hebei, China). Others, such as NKA-9 and AB-8, were purchased from Guangfu Fine Chemical Research Institute (Tianjin, China). The chemical and physical properties of these MARs were listed in Table 1.

### 3.2. Preparation of Crude Extracts from “Dahongpao” Tangerine

Crude extraction of PMFs was carried out according to our previous reports, with modifications [43,44]. The powdered peels (5.0 kg) were extracted with 89.1% ethanol solution (100 L). The miscible liquid was extracted for 34.1 min in ultrasound (300 W) at 40.9 °C and filtered at room temperature. The supernatant was collected, and the residue was extracted again with the same procedure. All the supernatants were combined and then concentrated in vacuo at 45 °C to give a crude extract of total PMFs at a concentration of 28 mg/mL.

### 3.3. Determination of PMF Contents

The crude extract was diluted 40-fold with 25% ethanol solution and then filtrated through a 0.22 μm hydrophilic polytetrafluoroethylene (PTFE) syringe filter. The content of nine kinds of PMFs was determined using UPLC-Q-TOF-MS/MS, and the sum of them was regarded as the content of total PMFs. Chromatographic separations were performed on a 2.1 × 100 mm, 1.7 μm ACQUITY UPLC BEH C18 column (Waters, UK). The mobile phase consisted of water/formic acid (99.99%: 0.01%, *v*/*v*) (A) and methanol (B) at a flow rate of 0.4 mL/min, the gradient profile was as follows: 0–0.6 min, 10–20% B; 0.6–5 min, 20–70% B; 5–7 min, 70–90% B; 7–9 min, 90% B, 9–11min, 10% B. The absorbance was measured at 330 nm for PMFs. The injection volume was 1.0 μL, and the temperature of the column was kept at 40 °C.

### 3.4. Static Adsorption and Desorption Tests

#### 3.4.1. Pretreatment of Macroporous Adsorptive Resins

Before the study, the resin was treated referred to the manufacturer’s instructions. The MARs were pretreated overnight by ethanol, in order to active the adsorbents and remove the porogenic agents and monomers, which were trapped in the pores in synthesis process. The resins were soaked by ethanol for 6 h, and then washed by water until the detergent was not cloudy. This step was repeated until no residues remained, and further washed by distilled water until the pH was 7.0.

#### 3.4.2. Static Adsorption and Desorption Properties of the Resins

Static adsorption and desorption tests were performed to select the proper macroporous resin for PMF separation. The tests were performed using the method reported by Toor et al. [45] with modifications. Briefly, 0.5 g (wet resin) tested resins were mixed with 40 mL sample solution (the initial concentrations of total PMFs were 0.7 mg/mL). After adsorption equilibrium was reached, the resins were washed by deionized water and then desorbed with 50 mL of 90% ethanol solution. The flasks were both shaken (130 rpm) at 25 °C for 12 h in adsorption and desorption tests.

The adsorption capacity, desorption capacity, and desorption ratio of the resins were calculated using the following equations:

(1) $$\;Q_{e}=\frac{(C_{o}-C_{e})\times V_{i}}{W},\;$$

(2) $$\;Q_{d}=\frac{C_{d}V_{d}}{W},\;$$

(3) $$\;D=\frac{C_{d}V_{d}}{(C_{o}-C_{e})V_{i}}\times 100\%,\;$$

where Q_e_ is the adsorption capacity (mg/g); C_0_ and C_e_ are the initial and equilibrium concentrations of PMFs in the solutions, respectively (mg/mL); V_i_ is the volume of the initial sample solution (mL); W is the dry weight of the tested resins (g); Q_d_ is the desorption capacity (mg/g); C_d_ is the concentration of solutes in the desorption equilibrium solution (mg/mL); V_d_ is the volume of the desorption solution (mL); D is the desorption ratio.

#### 3.4.3. Adsorption Kinetics of Selected Resin

The adsorption kinetics curve for total PMFs on the selected HPD 300 resin was studied by mixing 160 mL sample solutions with pre-weighed amounts of hydrated resin (2.0 g wet resin) in 500 mL flasks. The flasks were then shaken (130 rpm) at 25 °C for 24 h. The concentration of total PMFs in adsorption solution were determined at different time intervals. Adsorption kinetics are usually analyzed using three models: pseudo-first order, pseudo-second order, and intra-particle diffusion kinetics models. The models can be expressed by the following mathematical formulas:

(4) $$\;Q_{t}=Q_{e}-Q_{e}e^{k_{1}t},\;$$

(5) $$\;Q_{t}=\frac{k_{2}Q_{e}^{2}t}{1+k_{2}Q_{e}t},\;$$

(6) $$\;Q_{t}=k_{3}t^{\frac{1}{2}}+c,\;$$

where Q_e_ (mg/g) is the adsorptive capacity; Q_t_ (mg/g) is the concentration of solute per mass of adsorbent at time t; k_1_, k_2_, and k_3_ are the pseudo-first order, pseudo-second order, and intra-particle diffusion rate constants, respectively; c is a constant.

To investigate the desorption property for total PMFs on the selected HPD 300 resin, the static desorption kinetics curve was plotted, which was studied in accordance with the adsorption kinetics.

#### 3.4.4. Adsorption Isotherms on the Selected Resin

Adsorption isotherms indicate the specific effect of equilibrium concentration of adsorbate on its degree of accumulation on the surface of adsorbent at a certain temperature, which can reflect the interaction between adsorbent and adsorbate, as well as the process and mechanisms of adsorption. Langmuir and Freundlich equations are two most popular formulas widely applied in description of the mechanisms of adsorption.

The model of Langmuir can be expressed as follows:

(7) $$\;Q_{e}=\frac{Q_{m}K_{L}C_{e}}{1+K_{L}C_{e}}\;$$

The Langmuir Equation (7) was converted to the linearized form (8) with C_e_ and C_e_/Q_e_ as an independent variable:

(8) $$\;\frac{C_{e}}{Q_{e}}=\frac{1}{K_{L}Q_{m}}+\frac{C_{e}}{Q_{m}},\;$$

where Q_e_ and C_e_ are the same as described above; K_L_ is the association Langmuir constant; Q_m_ (mg/g) is the maximum adsorptive capacity, theoretically.

The Freundlich equation is applied under the condition of nonideal adsorption on a heterogeneous surface for liquid and gas phase adsorption with two main parameters (equilibrium concentration and adsorptive capacity) in many different adsorbate/adsorbent systems. 

This adsorption model can be expressed by the following equation:

(9) $$\;Q_{e}=K_{F}C_{e}^{\frac{1}{n}}.\;$$

Further, Equation (9) can be transformed to Equation (10) as follows:

(10) $$\;{\mathrm{lnQ}}_{e}={\mathrm{lnK}}_{F}+\frac{1}{n}{\mathrm{lnC}}_{e}.\;$$

### 3.5. Dynamic Adsorption and Desorption Tests

Dynamic adsorption and desorption tests were carried out in a laboratory-scale glass column (300 mm × 22 mm i.d.) packed with HPD 300 resin (11.0 g, wet resin). The bed volume (BV) was approximately 10 mL. To investigate the relationship between adsorption capacity and external factors (sample concentration, flow rate, and loading sample volumes), sample solutions with different concentrations (2.42, 5.45, 8.70, and 10.75 mg/mL) were carefully loaded onto the resin column at feeding speeds of 1.5, 2.5, 4.0, and 5.5 BV/h, respectively, and the loading volumes of the sample solutions varied from 1 to 21 BV.

After the column was saturated with PMFs, the column was washed with 50%, 70%, and 90% ethanol, with gradient modes at eluting speeds of 2.5, 4.0, and 5.5 BV/h, and the volumes of eluent varied from 1 to 24 BV. The desorbed fraction was concentrated in a rotary evaporator, and the content and recovery yield of total PMF were calculated.

### 3.6. Comparison of PMFs before and after Enrichment

The obtained PMFs in crude extract in Section 3.2 (before enrichment), and the eluent after the resin column (after enrichment) were separately rotary evaporated to remove the organic solvent and freeze-dried as powders. The above powders (0.5 g) were weighed, dissolved in methanol, and diluted to 25 mL. The flavonoids contents were determined according to the method in Section 3.3, and the total PMF contents were calculated.

A 0.5 g amount of the powders before and after enrichment were dissolved in different volumes of methanol, respectively, and they were formulated into two solutions with equal total PMF contents (total PMF contents were both 0.5 mg/mL). The color difference due to the other impurities was compared according to the method in Section 3.3.

### 3.7. Separation of PMFs by MAR

#### 3.7.1. Preparation of PMF Solutions

The purified lyophilized mixture of PMFs was weighed to 5.0 g, and dissolved with methanol into 100 mg/mL, and the solution was put into a 5 mL centrifuge tube through a 0.22 μm PTFE membrane for later experiments.

#### 3.7.2. Optimization of Experimental Conditions for Prep-HPLC

PMFs were separated and isolated on a MS-Directed AutoPure System (Waters, Milford, MA, USA), which was equipped with a 515 pump, a 2767 Sample Manager, a photodiode array (PDA) detector, and an ACQUITY QDa detector. The wavelength of PDA detector was set at 330 nm. The detection conditions were as follows: the ionization mode was ESI^+^, cone voltage 15 V, capillary voltage 0.8 kV, source temperature 100 °C, desolvation temperature 600 °C, cone gas flow 50 L/h, desolvation gas flow 600 L/h, the scan ranged from *m*/*z* 100 to *m*/*z* 1000, the scan time was 0.2 s, and the compensation pump flow 1.0 mL/min.

The injection concentration was 100 mg/mL. The effects of different preparation columns (Sunfire and XBridge) and the mobile phases (methanol-water; acetonitrile-water; methanol/acetonitrile-water with 0.1% formic acid; 0.1% acetic acid; and 0.1% ammonium formate), flow rate, elution gradient and injection volume for the separation of PMFs were separately investigated. Then the automatic collection of trigger conditions was optimized based on the optimal separation conditions.

#### 3.7.3 Identification of PMFs

The PMFs were rotary evaporated, respectively, to remove the organic solvent, and weighed after freeze-drying. The monomers were detected by the UPLC-Q-TOF-MS/MS method reported in prior study [43]. The mass spectrometry conditions were as follows: capillary voltage 0.45 kV, cone voltage 40 V, source temperature 100 °C, desolvation temperature 400 °C, cone gas flow 50 L/h, desolvation gas flow 600 L/h, low energy 6 V, and high energy ramp 20 to 40 V. TOF-MS ranged from *m/z* 100 to *m/z* 1000. The scan time was 0.2 s. ^1^H-NMR and ^13^C-NMR spectra were recorded in CDCl_3_ on an AVANCE III10600 Bruker NMR (Bruker Biospin Co., Ltd, Karlsruhe, Germany).

### 3.8. Statistical Analysis

Data were expressed as the means ± standard deviation of three replicates. Significant differences among eight resins (*p* < 0.05) were assessed by one-way analysis of variance (ANOVA), followed by Duncan’s multiple-comparison test (SPSS 22.0, IBM, Armonk, NY, USA). The results of adsorption kinetics and isotherms were analyzed and fitted using Origin 8.0 and Excel. UPLC-Q-TOF-MS/MS data was processed by UNIFI 1.7 software and NMR data was processed using MestReNova 9.0.1.

## 4. Conclusions

In this paper, we report a new method for isolation and purification of citrus PMFs using macroporous adsorption resins combined with prep-HPLC. HPD 300 resin was selected to enrich target compounds and remove impurities, and prep-HPLC was used to isolate and purify PMFs. Using the present method, five PMFs, including 5,6,7,4′-tetramethoxyflavone, nobiletin, tangeretin, sinensetin, and 5-hydroxy-6,7,8,3′,4′-pentamethoxyflavone, could be simultaneously obtained from the peel extract of “Dahongpao” tangerine, and the purity of the PMFs are all above 95%. Our study showed that the MS-directed prep-HPLC is a highly efficient technique for separation and purification of citrus PMFs and, potentially, other citrus flavonoids.

## Figures and Tables

**Table 1:** Physical properties of macroporous resins used.

MAR Type	Polarity	Particle Diameter (mm)	Surface Area (m^2^/g)	Average Pore Dimeter (Å)
D 101	Non-polar	0.315–1.25	650–700	100–110
HPD 100	Non-polar	0.30–1.25	650–700	85–90
AB-8	Weak polar	0.30–1.25	480–520	130–140
HPD 300	Weak polar	0.30–1.25	800–870	50–55
HPD 400	Middle polar	0.30–1.25	500–550	75–80
DM 130	Middle polar	0.30–1.25	500–550	90–100
NKA-9	Strong polar	0.30–1.25	250–290	155–165
HPD 600	Strong polar	0.30–1.20	550–600	80

**Table 2:** The adsorption and desorption characteristics of total polymethoxylated flavones (PMFs) on eight macroporous resins.

MAR Type	Adsorption Capacity (mg/g)	Desorption Capacity (mg/g)	Desorption Rate (%)
D 101	227.51 ± 5.87 ^c^	194.59 ± 19.49 ^c^	85.45 ± 6.36% ^ab^
HPD 100	271.52 ± 9.60 ^b^	248.17 ± 4.01 ^b^	91.43 ± 1.75% ^a^
AB-8	275.13 ± 3.67 ^b^	241.59 ± 1.51 ^b^	87.82 ± 1.73% ^ab^
HPD 300	301.75 ± 3.32 ^a^	274.01 ± 0.40 ^a^	90.81 ± 0.83% ^a^
HPD 400	265.57 ± 2.71 ^b^	239.17 ± 2.51 ^b^	90.06 ± 0.03% ^a^
DM 130	236.29 ± 1.82 ^c^	200.88 ± 2.66 ^c^	85.02 ± 1.78% ^ab^
NKA-9	209.03 ± 2.04 ^d^	173.06 ± 0.65 ^d^	82.80 ± 1.12% ^b^
HPD 600	238.03 ± 5.77 ^c^	206.98 ± 2.72 ^c^	87.00 ± 3.25% ^ab^

The different letters (^a–d^) between the same column represent a significant difference between the different resins (*p* < 0.05).

**Table 3:** Adsorption kinetics modeling and parameters for PMFs on HPD 300 resin.

Models	Equations	Parameters	Values
Pseudo-first order model	ln(175.2235-Q_t_) = 5.1661 − 5.5651t	R^2^	0.7920
		Q_e_ (mg/g)	175.2235
		k_1_ (L/min)	5.5651
Pseudo-second order model	t/Q_t_ = 0.0054t + 0.0007	R^2^	0.9999
		Q_e_ (mg/g)	185.1852
		k_2_ (g/(mg min))	0.0417
Intra-particle diffusion model	$Q_{t}=75.147t^{\frac{1}{2}}+73.414$ (first stage)	R^2^	0.9660
		k_3_ (mg/(min^1/2^·g))	75.147
		C(mg/g)	73.414
	$Q_{t}=2.2816t^{\frac{1}{2}}+174.45$ (second stage)	R^2^	0.6008
		k_3_(mg/(min^1/2^·g))	2.2816
		C(mg/g)	174.45

**Table 4:** Langmuir and Freundlich parameters of PMFs on HPD 300 resin at different temperatures.

Models	Equations	T (°C)	Parameters				
			R^2^	K_L_	Q_m_	K_F_	1/n
Langmuir	Ce/Qe = 0.00010Ce + 0.00425	25	0.9710	44.1981	235.1302	-	-
	Ce/Qe = 0.00017Ce + 0.00448	35	0.9845	26.6570	223.2240	-	-
	Ce/Qe = 0.00021Ce + 0.00537	45	0.9889	26.1808	186.2802	-	-
Freundlich	Qe = 314.5Ce0.2790	25	0.9637	-	-	314.5000	0.2790
	Qe = 281.7663Ce0.3091	35	0.9473	-	-	281.7663	0.3091
	Qe = 224.44Ce0.2927	45	0.9251	-	-	224.4400	0.2927

**Table 5:** Composition and content of PMFs of tangerine peel extracts before and after HPD 300 resin purification.

No.	Contents	Before Enrichment (mg/g)	After Enrichment (mg/g)	Increased Times
1	Isosinensetin	1.239 ± 0.046	20.59 ± 1.65	16.62
2	Sinensetin	1.913 ± 0.066	37.56 ± 3.04	19.63
3	5,7,3′,4′-Tetrathoxyflavone	0.056 ± 0.001	1.92 ± 0.60	34.29
4	5,6,7,4′-Tetrathoxyflavone	1.207 ± 0.113	24.06 ± 3.79	19.93
5	Nobiletin	19.27 ± 0.743	355.09 ± 18.45	18.43
6	3,5,6,7,8,3′,4′-Hetamethoxyflavone	0.554 ± 0.021	12.14 ± 1.56	21.91
7	5,7,4′-Trimethoxyflavone	0.019 ± 0.005	1.67 ± 0.16	87.89
8	5-Hydroxy-6,7,8,3′,4′-pentamethoxyflavone	1.555 ± 0.063	30.27 ± 2.57	19.47
9	Tangeretin	5.908 ± 0.229	111.27 ± 7.10	18.83
10	Total PMFs	31.720 ± 1.255	594.57 ± 28.05	18.74

**Table 6:** The PMFs standards used in this study.

Contents	CAS No.	Molecular Formula	Monoisotopic Mass (g/mol)	Purity
Isosinensetin	17290-70-9	C_20_H_20_O_7_	372.1209	>98.0%
Sinensetin	2306-27-6	C_20_H_20_O_7_	372.1209	≥98.0%
5,7,3’,4’-Tetrathoxyflavone	855-97-0	C_19_H_18_O_6_	342.1103	≥97.0%
5,6,7,4’-Tetrathoxyflavone	1168-42-9	C_19_H_18_O_6_	342.1103	≥98.0%
Nobiletin	478-01-3	C_21_H_22_O_8_	402.1315	≥95.0%
3,5,6,7,8,3’,4’-Hetamethoxyflavone	1178-24-1	C_22_H_24_O_9_	432.1420	>98.0%
5,7,4’-Trimethoxyflavone	5631-70-9	C_18_H_16_O_5_	312.0998	≥98.7%
5-Hydroxy-6,7,8,3’,4’-pentamethoxyflavone	2174-59-6	C_20_H_20_O_8_	388.1158	≥98.0%
Tangeretin	481-53-8	C_20_H_20_O_7_	372.1209	≥95.0%

## Supplementary Materials

The following are available online. Figure S1: ^13^C- and ^1^H-NMR spectrum of 5,6,7,4’- tetramethoxyflavone, Figure S2: ^13^C- and ^1^H-NMR spectrum of nobiletin, Figure S3: ^13^C- and ^1^H-NMR spectrum of tangeretin, Figure S4: ^13^C- and ^1^H-NMR spectrum of sinensetin, Figure S5: ^13^C- and ^1^H-NMR spectrum of 5-hydroxy-6,7,8,3’,4’-pentamethoxyflavone.
