# Supplementary material for: Simultaneous Separation and Purification of Five Polymethoxylated Flavones from “Dahongpao” Tangerine (Citrus tangerina Tanaka) Using Macroporous Adsorptive Resins Combined with Prep-HPLC
Source: Molecules. 2018 Oct 16;23(10):2660. doi: 10.3390/molecules23102660 (PMC6222627; doi:10.3390/molecules23102660)
Supplement: Supplementary file 1 [file molecules-23-02660-s001.pdf]

Supplementary Materials:

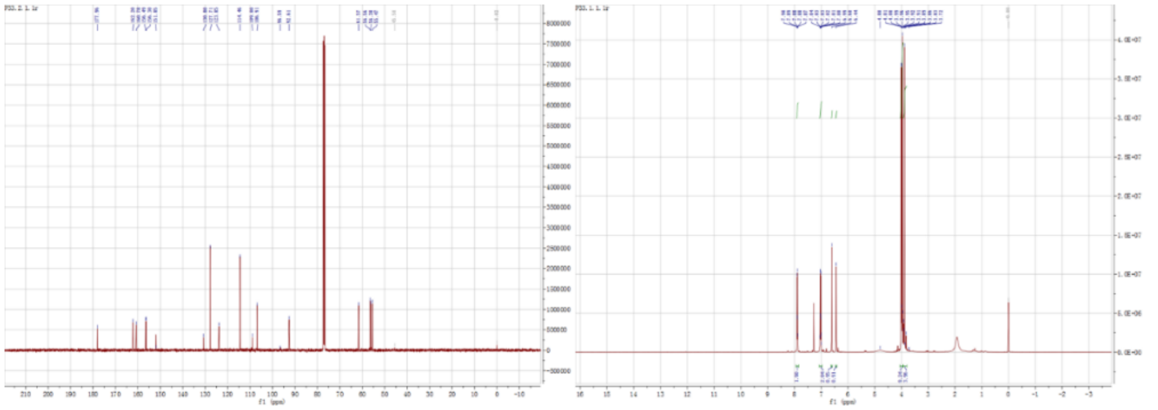

Figure S1. ^{13}C- and ^{1}H-NMR spectrum of 5,6,7,4′-tetramethoxyflavone.

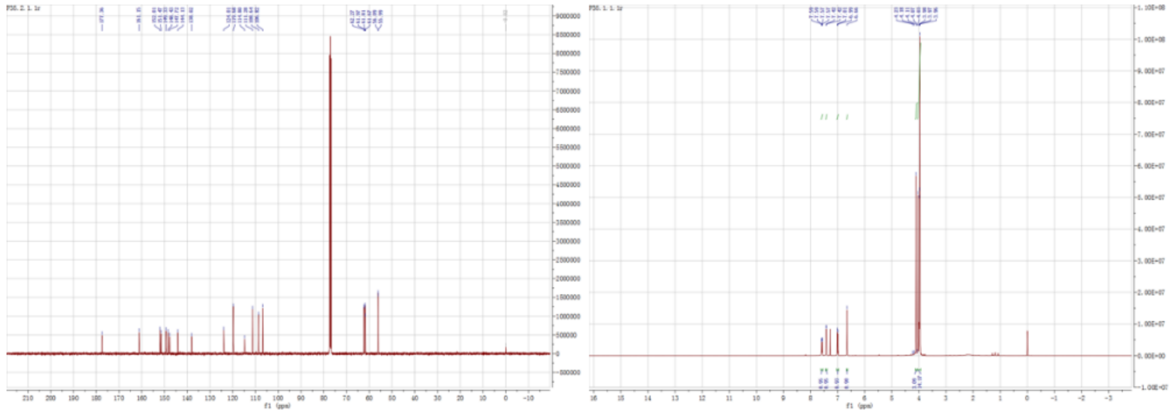

Figure S2. ^{13}C- and ^{1}H-NMR spectrum of nobiletin.

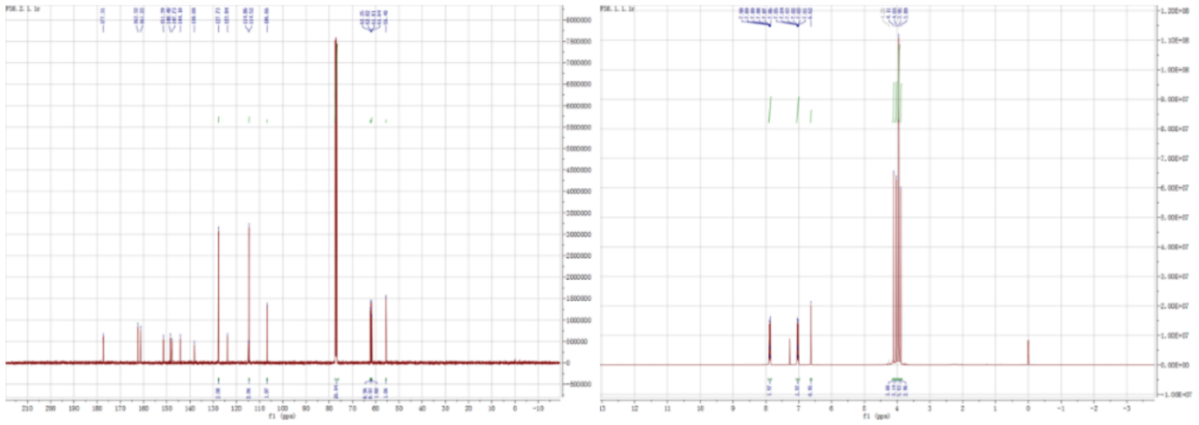

Figure S3. ^{13}C- and ^{1}H-NMR spectrum of tangeretin.

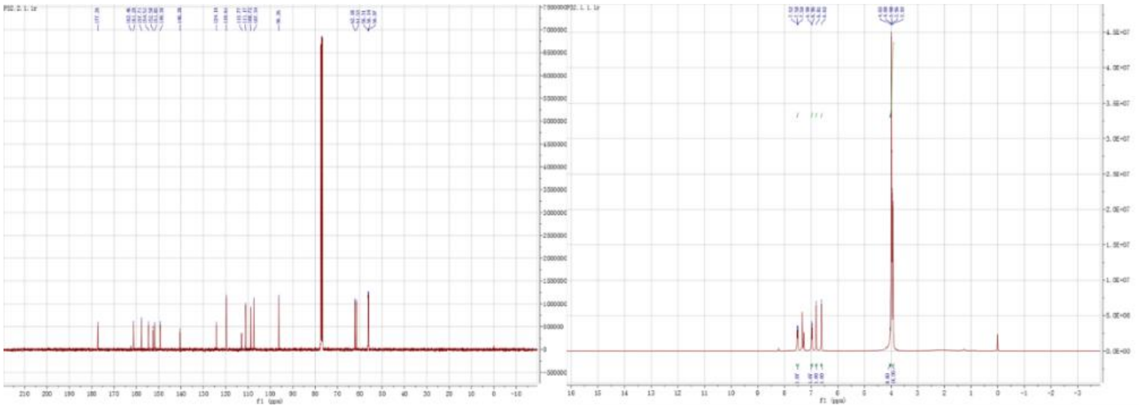

Figure S4. ^{13}C- and ^{1}H-NMR spectrum of sinensetin.

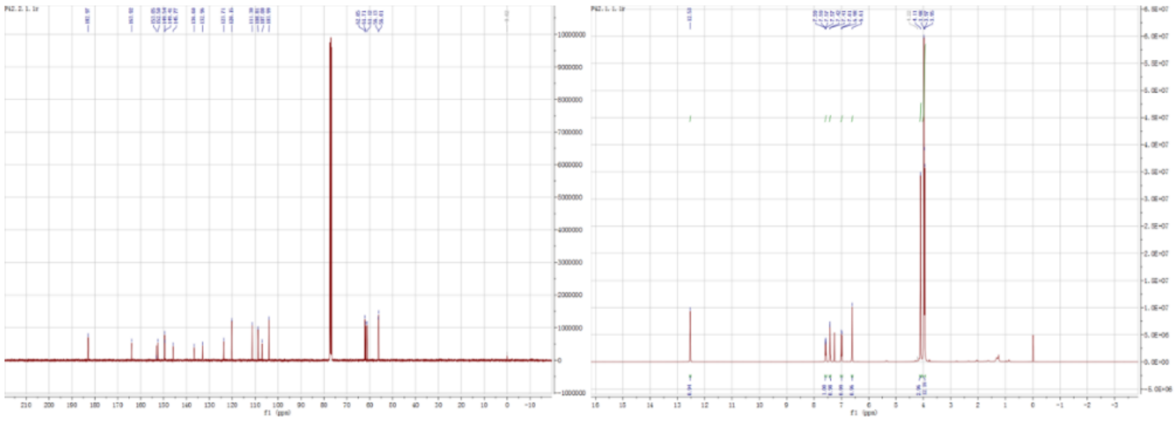

Figure S5. ^{13}C- and ^{1}H-NMR spectrum of 5-hydroxy-6,7,8,3′,4′-pentamethoxyflavone.
